# Association of smokeless tobacco with oral cancer: A review of systematic reviews

**DOI:** 10.18332/tpc/112596

**Published:** 2019-10-08

**Authors:** Smita Asthana, Parul Vohra, Satyanarayana Labani

**Affiliations:** 1 National Institute of Cancer Prevention and Research, Indian Council of Medical Research, India

**Keywords:** meta-analysis, systematic reviews, smokeless tobacco, oral cancer

## Abstract

**INTRODUCTION:**

Various primary studies and systematic reviews have been conducted to explain the association between smokeless tobacco and oral cancer. This study aims to consolidate and summarize the risk estimates from various systematic reviews with or without meta-analysis to provide the spectrum of estimates on the association between smokeless tobacco use and oral cancer.

**METHODS:**

A comprehensive literature search was done on various databases (PubMed, Google Scholar, IndMED, and TOXLINE) by two of the authors independently. Both qualitative and quantitative data extraction and analysis were performed for the included systematic reviews. Range of risk estimates was obtained and analyzed as quantitative findings due to the limitation of an overview of reviews for the pooled estimates. CASP (Critical Appraisals Skills Programme) and AMSTAR 2 (A Measurement Tool to Assess Systematic Reviews) tools were used for the quality assessment of the studies included.

**RESULTS:**

In total, 12 systematic reviews with or without meta-analysis were included in the review. There was a positive and strong association of Smokeless Tobacco (SLT) use with oral cancer irrespective of gender, region, and type of smokeless tobacco. The risk estimate for the South–East Asia Region (SEAR) ranged 4.44–7.90, for Gutkha it was 8.67, while for Paan it ranged 6.3–7.90 and for overall SLT it ranged 1.36–7.90. Risk estimate for females ranged 5.83–14.56.

**CONCLUSIONS:**

The study confirmed the association between SLT use and oral cancer. These findings are of high importance, especially to the South-East Asia Region.

## INTRODUCTION

Smokeless tobacco (SLT) is a type of tobacco that is not burnt but consumed in raw form. It is also known as chewing tobacco, oral tobacco, spit or spitting tobacco, dip, chew, and snuff^1^. Globally, there are many SLT products available, and these can be in the form that can be either chewed or snuffed orally or nasally, or applied over the teeth and gums, gargled, or drunk^2^. It has been estimated that >90% of the world’s SLT users live in the South–East Asia Region (SEAR). A large proportion of these users come from India, which has the dubious distinction of being one of the largest producers and consumers of SLT. India is the third largest producer of tobacco after China and Brazil^3^. In spite of the adoption of the WHO Framework Convention on Tobacco Control (WHO FCTC) and the implementation of Cigarette and Other Tobacco Products Act (COTPA), SLT use has steadily increased in India from 19% in 1998 to 25% in 2010, among persons aged 15–49 years^4-6^.

In 2007, the World Health Organization noted that SLT contributes significantly to the overall world tobacco problem^7^. People in many regions and countries, including North America, northern Europe, India, other Asian countries apart from India, and parts of Africa have a long history of using SLT products^1^. Wet snuff is a form of SLT used in the Western world. Nasal snuff is a dry powder known as nas or naswar, used in the SEAR and EMR (East Mediterranean Region) WHO regions^2^. SLT products contain carcinogenic chemicals^8^. Approximately 28 chemical constituents present in SLT are carcinogens. These are primarily from 3 groups of compounds: nonvolatile, alkaloid-derived TSNAs; N-nitrosoamino acids; and volatile N-nitrosamines. Among these carcinogens, researchers have identified nitrosamine as the most prominent carcinogen^9^. The World Health Organization has classified SLT products as human carcinogenic compounds, in particular, tobacco-specific nitrosamines, which account for 76–91% of the total N-nitroso compound (NOC) burden^10^.

Oral cancer belongs to a larger group of cancers called head and neck cancers. Such cancers develop mostly in the squamous cell linings of the mouth, tongue, and lips. It is the cancer of the lining of the lips, mouth, or upper throat^11^. Oral cancer^12^ has a 5-year survival rate of 65.3%, which may vary widely depending on factors such as stage of cancer, age of patient, extent of tumor location of the disease in the mouth etc. Regional incidence varies with the highest rates in South Asia, particularly India, Bangladesh, Sri Lanka, Pakistan, and Afghanistan^13^. Oral cancer is the third most common form of cancer in India^14^ with over 52000 deaths in 2012 and a 2.3:1 male to female ratio. According to GLOBOCAN report 2018, a total of 119992 new cases were reported in India for both males and females with 92011 for males and 27981 for females. Oral cancer was also reported as one of the most prevalent cancers and the second leading cause of mortality in India^15^.

There is diversification among different systematic reviews based on regions of the world, with subgroups considered and outcomes evaluated in terms of effect measures. In addition, there are wide quality considerations in different systematic reviews in terms of variations in Risk of Bias (ROB) assessments. A segregated view, critical analysis, and summarization of effect measures of association between SLT and oral cancer from different global systematic reviews allow us to assess the problem in an overarching review. Thus, we aim to consolidate the data from various systematic reviews with or without meta-analysis and provide the spectrum of risk on the association between SLT and oral cancer, useful to public health researchers and policymakers.

## METHODS

### Literature search

Two of the researchers conducted independent literature searches. Searches were conducted in PubMed, Google Scholar, IndMED, and TOXLINE. The search strategy used, with the restriction to review, systematic reviews and meta-analysis, was:

[SLT OR oral tobacco OR non-burn tobacco OR snus OR gutkha OR *naswar* OR chew* tobacco OR tobacco powder OR tobacco tooth powder OR tobacco paste OR creamy snuff OR *mishri* OR *masher* OR dip tobacco OR tobacco water OR *tuibur* OR *hidakphu* OR *gul* OR *gutkha* OR *mawa* OR *khaini* OR *snuff* OR pan masala OR pan masala with tobacco OR *paan* OR pan with tobacco OR *zarda* OR *tambaku* OR betel quid tobacco OR betel tobacco OR tobacco flakes OR tobacco leaf OR dried tobacco OR *hogesoppu* OR *gnudi* OR *Kadapa* OR *Mainpuri* tobacco OR qiwam OR *kimam* OR dohra OR raw tobacco] AND [oral cancer OR oral carcinoma* OR oral malignant* OR oral tumour OR oral growth].

Various combinations of the keywords were also used in Google Scholar and other databases, such as, ‘SLT’, ‘oral cancer’, ‘oral neoplasm’, ‘systematic review’, and ‘meta-analysis’. Articles found were screened independently by two of the authors, on the basis of the inclusion and exclusion criteria. Papers cited in the references of the selected reviews were also examined. Selection of the reviews was made independently by two of the authors, and any conflict was resolved by deliberation with the third author.

### Inclusion criteria

The review included Systematic reviews and meta-analysis on observational studies (case-control and cohort) involving people with oral cancer in relation to SLT use. Included systematic reviews needed to have a qualitative conclusion on the topic along with sufficient quantitative data, including risk estimates in terms of odds ratio or relative risk for the assessment of included studies. Only those systematic reviews that were conducted in accordance with PRISMA (Preferred Reporting Items for Systematic reviews and Meta-Analysis) guidelines were included^16^.

There were no limitations on gender, age, and country of included participants in the review. There were no restrictions on publication date and region of the reviews. Among those systematic reviews that included various cancers, only results for oral cancers were considered.

### Exclusion criteria

Literature reviews, case series, case reports, and other primary studies, reviews published in a language other than English, and systematic reviews that included cross-sectional studies were excluded. Systematic reviews not done according to the PRISMA guidelines were also excluded.

### Data collection and reporting

Two independent researchers searched for related systematic reviews and meta-analysis in various databases. Relevant titles and abstracts were screened. Full-text articles were identified and assessed for the inclusion and exclusion criteria. Qualitative extraction included objective of review, year of publication, place of review, details of participants, study exposure, name and number of databases searched, confounders, publication date range of included studies, number, type, place of studies included, tool to appraise studies (odds ratio/relative risk), and method of analysis used in review. Quantitative extraction included data on odds ratio/relative risk for the pooled studies with variations on the basis of gender, WHO region, area of study, level of heterogeneity, and bias analysis. Estimates of risk from different systematic reviews were included in the form of a range of study variables. Data extraction was done independently by two authors, and any conflict was resolved by deliberation with the third author.

### Statistical analysis

Various summaries for qualitative measures are presented and conclusions drawn based on the findings. Quality assessment of included reviews was performed through the CASP and AMSTAR 2 tool. Quantitative analysis included a range of odds ratio/relative risk for various study variables such as SLT type-specific, gender-specific, region-specific, and study type-specific. RevMan 5.3 package was used to create forest plots. The protocol of this review was registered with PROSPERO Reg. no. CRD42019127796.

### Risk of bias

There are two tools for the qualitative assessment of systematic reviews, CASP and AMSTAR 2^17,18^. In CASP for items 1–5, 8–10 responses can be yes, no, or can’t tell; while in items 6 and 7 response can be yes, no, and not applicable if meta-analysis is not conducted. Similarly, in AMSTAR 2, the decision of yes, partial yes, or no is applied on different items of the tool^18^. According to the decision rules suggested by Shea et al.^19^ for AMSTAR 2, the reviews are given the ratings of high, moderate, low, or critically low.

## RESULTS

**Figure 1:**
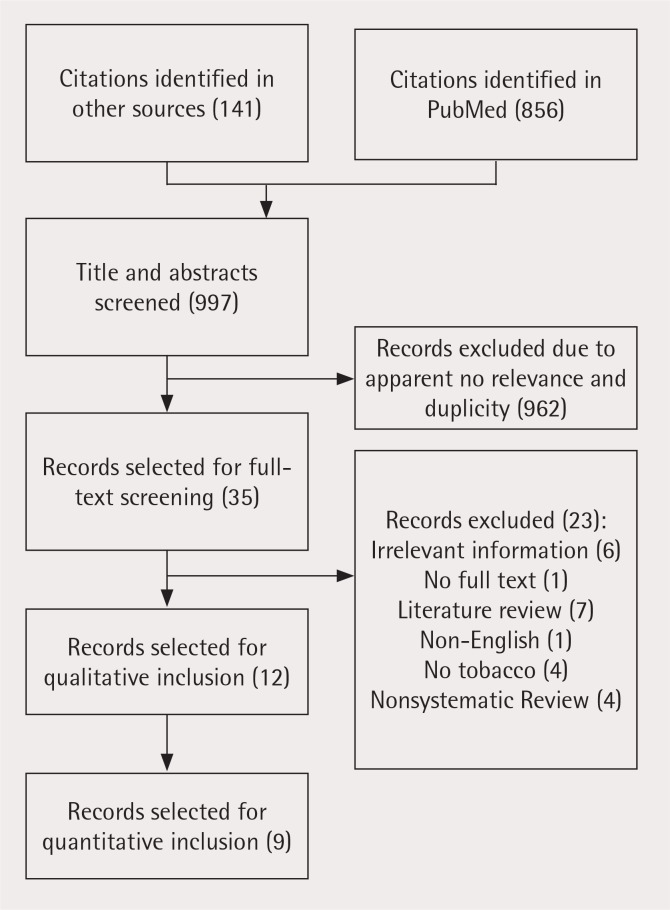
PRISMA flowchart for association of smokeless tobacco and oral cancer – A review of systematic reviews

In total, 856 articles were retrieved from PubMed when the search strategy with the restriction on systematic reviews and meta-analysis was applied. Another 141 articles were retrieved from various databases such as Google Scholar, Research Gate, IndMED and TOXLINE resulting in a total of 997 articles. These articles were then screened for the relevance of their title and abstract, and 962 articles were removed due to duplicity and non-relevance, resulting in 35 articles for full-text review. Of these, 23 were excluded due to various reasons such as non-systematic review, only literature review, insufficient information, not in English etc. Twelve articles selected were identified as relevant on the basis of the inclusion criteria (Figure 1), and which investigated the association between SLT and oral cancer risk, from different parts of the world^20-31^. Some of the published reviews could not be included in the study because of potential risk of bias due to non-strict criteria of inclusion of primary studies in the review and for not following PRISMA guidelines^32-34^. Two systematic reviews presented global estimates on the risk of developing oral cancer by SLT use^20,27^, while five reviews made the same estimates for India. There were three reviews that estimated the risks for cohort studies^20,21,24^ and four provided the risks for a combination (case-control and cohort)^20,25,27,28^. Some systematic reviews estimated SLT type-specific risk from various studies. Five reviews^20,22,24,26,28^ estimated the risk for SLT, whereas three estimated for chewing tobacco^20,23,26^ and risk estimates for paan/betel quid with tobacco were given in four reviews^20,21,23,25^. Separate estimates for males and females were given in three reviews^20,21,28^.

Two were only systematic reviews and thus were included only in the qualitative analysis, and one, due to low quality, was not included in the quantitative analysis. Therefore, nine studies that performed a systematic review and meta-analysis were included in both the qualitative and quantitative analyses.

### Qualitative analysis

Various descriptive information of the included systematic reviews were analyzed (Supplementary Table 1). Twelve reviews were selected on the basis of the inclusion and exclusion criteria, of which ten were systematic reviews with meta-analysis and two were only systematic reviews. Only three reviews gave estimates for the global population and two were exclusively for India (Supplementary Table 1). When the type of SLT use was considered, there were only three reviews that talked about SLT (in general) and nine considered one or more types of SLT (Table 1). Only three reviews gave separate risk estimates for males and females, and six gave combined estimates (Table 2). Reviews included more case-control studies than cohort studies (12 reviews, of which 4 were India-based).

**Table 1:** List of studies included for separate analysis regarding SLT type-specific association with oral cancer

*Citation details*	*Publication date range of studies included*	*Number of studies included*	*Model used*	*Odds Ratio OR (95% CI)*	*Relative Risk/Risk Ratio*	*Bias analysis Yes/No*	*Population Attributable Fraction (PAF) (95% CI)*	*Heterogeneity (I^2^) and p-value*
Asthana et al.^20^ (2019)	1960–2016	37	Odds ratio (random effects), heterogeneity, publication bias	Chewing 4.37 (3.27–5.83) Non-chewing 1.56 (1.04–2.36)	-	Done (funnel plot)	Not calculated	98% <0.001 79% <0.001
Asthana et al.^20^ (2019)	1960–2016	37	Odds ratio (random effects), heterogeneity, publication bias	Paan tobacco/areca nut+lime+tobacco 7.18 (5.48–9.41) Oral snuff 4.18 (2.37–7.38) Snus/moist snuff 0.86 (0.58–1.29) Gutkha 8.67 (3.59–20.95) Mainpuri 3.32 (1.32–8.36) Nasal snuff/dipping 1.20 (0.80–1.81) Unspecified/mixed 2.63 (1.73–4.00)	-	Done (funnel plot)	Not calculated	75% <0.001 44% 0.17 0.11% 0.88 62% 0.11 97% <0.001 66% 0.03 96% <0.001
Guha et al.^21^ (2014)	1933–2013	50	Meta relative risk using a random effects model, heterogeneity, PAF		Betel quid 6.19 (4.16–9.21)	Done (funnel plot)	Calculated	89.8%
Gupta et al.^22^ (2014)	Case-Control 1959–2012 Cohort 2008–2011	19	Adjusted odds ratio with 95% CI using crude effect, heterogeneity using Higgins’ H and I^2^ statistics, funnel plots and Egger’s test were used to evaluate publication bias	Smokeless Tobacco Case-Control Studies 7.46 (5.86–9.50)	Cohort 5.48 (2.57–11.71)	Done (Begg’s test, funnel plot)	Not calculated	75.03% <0.001 80.445% <0.001
Khan et al.^23^ (2014)	1989–2013	21	Odds ratio (with inverse variance method using fixed and random effect method), heterogeneity	Chewing tobacco 4.3 (3.1–5.8) Paan/Betel quid with tobacco 6.3 (3.9–10.2)	-	Not done	Not calculated	NA
Lee et al.^24^ (2009)	Systematic review and meta-analysis	89	Odds ratio (random effects), heterogeneity, publication bias	Any smokeless tobacco 1.36 (1.04–1.77) Snuff (Scandinavia) 0.97 (0.68–1.37)	-	Done (funnel plot)	Not calculated	74.1% 58.8%
Petti et al.^25^ (2013)	1989–2011	14	Pooled odds ratio	Betel quid 7.90 (6.71–9.30)	-	Done (funnel plot)	Not calculated	-
Prasad & Dahr^26^ (2018)	1971–2015	22	Random effect odds ratio, heterogeneity	6.59 (5.18-8.39)	-	Not done	Not calculated	74.9% 0.001
Siddiquiet al.^27^ (2015)	1952–2012	33	Random effect odds ratio, heterogeneity	Total 3.43 (0.70–1.28)	-	Not done	Not calculated	0% <0.001
Sinha et. al.28 (2016)	1955–2015	25	Odds ratio using the random effect model, heterogeneity test using I2 statistics, publication bias	5.55 (5.07–6.07)	-	Done (funnel plot, Egger’s test, Begg-Mazumdar’s test)	0.60 (0.57–0.63)	95% <0.001

**Table 2:** List of studies included for separate analysis regarding gender-specific association* with oral cancer

*Citation details*	*Publication date range of studies included*	*Number of studies included*	*Model used*	*Odds Ratio OR (95% CI)*	*Bias analysis Yes/No*	*Population Attributable Fraction (PAF) (95% CI)*	*Heterogeneity (I^2^) and p-value*
Asthana et al.^20^ (2019)	1960–2016	37	Odds ratio (random effects), heterogeneity, publication bias	Males 2.72 (1.73–4.27) Females 5.83 (2.93–11.58) Both 3.35 (2.34–4.78)	Done (funnel plot)	Not calculated	98% <0.001 97% <0.001 87% <0.001
Guha et al.^21^ (2014)	1933–2013	50	Meta relative risk using the random effects model, heterogeneity, PAF	Males 5.37 (3.91–7.36) Females 14.56 (7.63–27.76) Both 9.64 (5.96–15.58)	Done (funnel plot)	44.7% 63.2% 49.5%	88.7% 93.7% 96.8%
Petti et al.^25^ (2013)	1989–2011	14	Pooled odds ratio	Indian Subcontinent 7.90 (6.71–9.30)	Done (funnel plot)	Not calculated	NA
Prasad & Dahr^26^ (2018)	1971–2015	22	Random effect odds ratio, heterogeneity	Case-control 6.59 (5.18–8.39)	Not done	Not calculated	74.9% 0.001
Siddiqui et al.^27^ (2015)	1952–2012	33	Random effect odds ratio, heterogeneity	Total 3.43 (0.70–1.28)	Not done	Not calculated	0% <0.001
Sinha et. al.^28^ (2016)	1955–2015	25	Odds ratio using a random effect model, heterogeneity test using I^2^ statistics, publication bias	Both 5.85 (5.29– 6.48) Males 5.16 (4.49– 5.94) Females 12.03 (9.49– 15.25) -	Done (funnel plot, Egger’s test, Begg-Mazumdar’s test)	Not calculated	95% <0.001

* No Relative Risk/Risk Ratio given for gender-specific association with oral cancer.

CASP and AMSTAR 2 tools were used for the quality assessment of included systematic reviews. The scoring of each systematic review, according to the CASP tool, is given in Supplementary Table 3. According to the CASP tool, most of the reviews had maximum scores, except for two systematic reviews because they lacked a meta-analysis. Two of the included systematic reviews performed quality assessments. Assessment using AMSTAR 2 was performed for different items of the tool that affected the quality of reviews (Supplementary Table 4). Most of the reviews could not account for risk of bias, with a lack of bias analysis and its interpretation in their results. Only 5 reviews performed the bias analysis and interpreted the effect of bias in their results.

### Quantitative analysis

**Figure 2:**
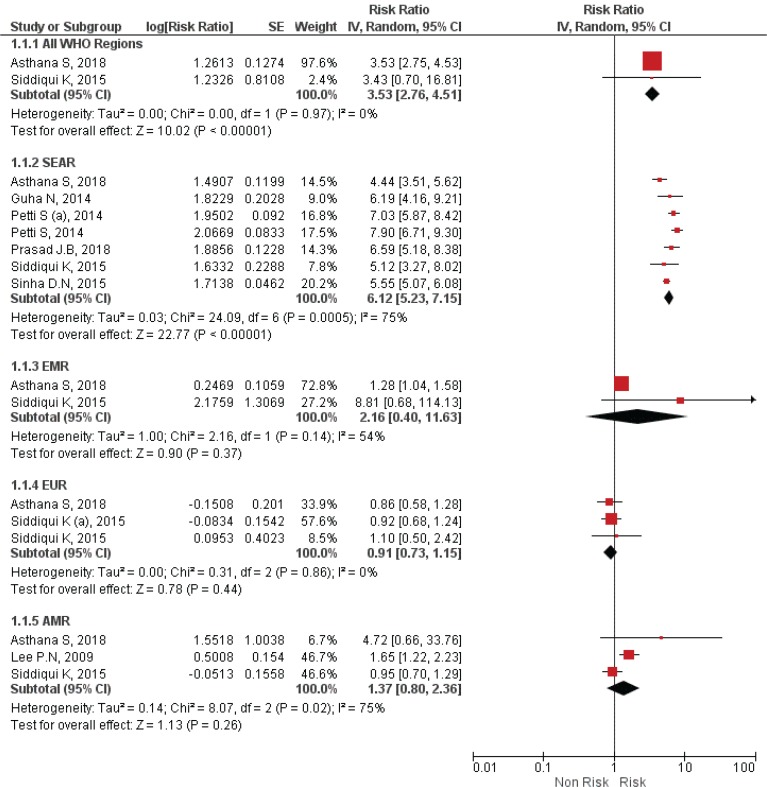
Forest Plot for smokeless tobacco use in the development of oral cancer by WHO regions by random effects model. SEAR: South East Asian region. EMR: East Mediterranean Region. EUR: European region. AMR: American Region.

When all WHO regions were considered, only two systematic reviews gave a high odds ratio indicating a high risk of association between SLT use and oral cancer. The odds ratio for the global population was 3.5 (95% CI: 2.75–4.51) (Figure 2). Other WHO regions such as SEAR, AMR (American Region), EUR (European Region), EMR, and WPR (Western Pacific Region) were also considered. Six studies were performed for the SEAR region, and odds ratios ranged from 4.44 (95% CI: 3.51–5.62) to 7.90 (95% CI: 6.71–9.30), the highest globally. The risk estimates for EUR showed the least risk of oral cancer associated with SLT with OR values ranging 0.85–1.10. EMR and AMR had higher odds ratios than EUR, but there exists a considerable difference compared with SEAR. A review that obtained estimates for WPR gave an odds ratio of 15.03 (95% CI: 9.87–22.8) for Taiwan.

**Figure 3:**
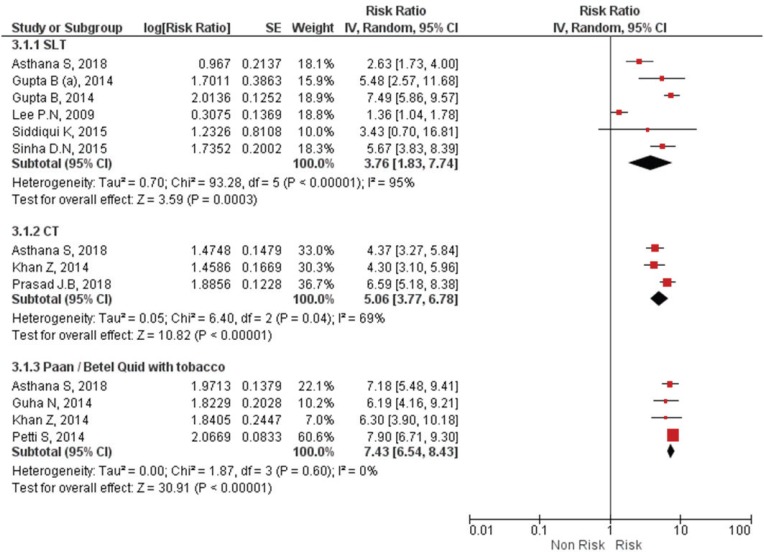
Forest Plot for smokeless tobacco use in the development of oral cancer by SLT type by random effects model. SLT: Smokeless Tobacco. CT: Chewing Tobacco.

Table 1 and Figure 3 describe the list of all the studies that gave risk estimates on type-specific SLT. Five of the included reviews analyzed SLT as a whole, and six for a specific type of SLT. Odds ratio for SLT ranged from 1.36 (95% CI: 1.04–1.77) to 7.49 (95% CI: 5.86–9.50), with Relative Risk (RR) from 2.63 to 5.48. Odds ratio for Gutkha was the highest with an odds ratio of 8.67 (95% CI: 3.59–20.95). Oral snuff, nasal snuff and moist snuff/snus had a huge disparity in the OR, ranging from 4.18 (95% CI: 2.37–7.38), 1.20 (95% CI: 0.80–1.81) and 0.86 (95% CI: 0.58–1.29), respectively, but still of less risk than chewing tobacco.

**Figure 4:**
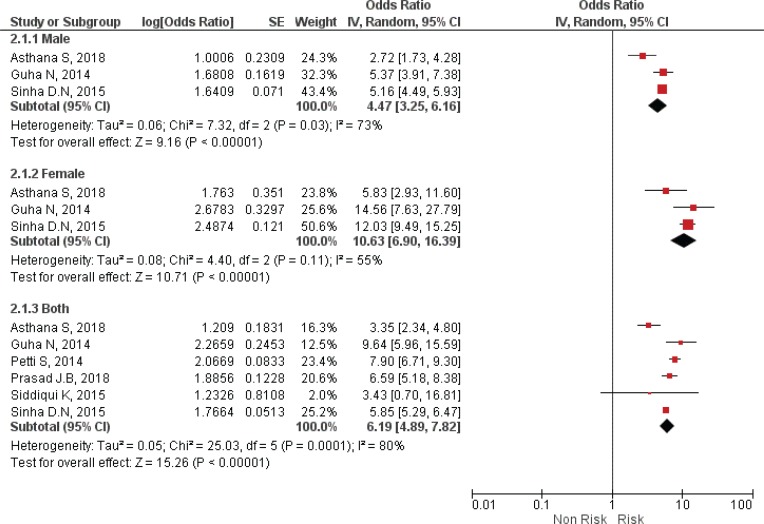
Forest Plot for smokeless tobacco use in the development of oral cancer by Gender, by random effects model.

Separate analysis for males and females was performed by only three reviewers, while six performed analysis for males and females combined (Table 2 and Figure 4). Analysis has shown that females have a higher risk of oral cancer than males, due to SLT use, with odds ratios ranging from 5.83 (95% CI: 2.93–11.60) to 14.56 (95% CI: 7.63–27.79). The range of OR for males and females combined was from 3.35 (95% CI: 2.34–4.78) to 9.64 (95% CI: 5.96–15.58).

Researchers mainly included case-control studies in their reviews, with cohort and a combination of both also included (Supplementary Table 2). Four of the reviews included case-control studies with OR ranging from 3.66 (95% CI: 2.83–4.74) to 7.23 (95% CI: 4.96–10.56). One review performed a pooled analysis of case-control studies in the period 1920–1988 and after 1990, separately. The risk estimates were less for the studies conducted after 1990. Range of relative risk for the cohort studies was less than for the case-control studies. When reviewers performed a pooled analysis for both types of studies collectively, the OR ranged from 3.43 (95% CI: 0.7– 1.28) to 7.90 (95% CI: 6.71–9.30).

## DISCUSSION

We consolidated and summarized the data from various systematic reviews and meta-analysis that evaluated the association between SLT and oral cancer. In contrast to individual effect measures from different systematic reviews, a comprehensive overview of the association between oral cancer and consumption of SLT was presented in the present review. The risk estimates varied for different types of SLT and also for gender, while region-specific differentials exist. The associated risk in females was higher than in males. Specific types of SLT such as Gutkha, chewing tobacco, and paan/betel quid, with or without tobacco, posed a more significant threat.

Global risk estimates for all WHO regions were provided by only two reviews. Both had high-risk estimates of more than 3-fold risk^20,27^. The estimates of Siddiqi et al.^27^, though high, were non-significant, the reason might be that data were limited to those countries and diseases for which reliable prevalence and risk data were available, hence the authors might have excluded some critical studies. As the primary objective of this review was to examine the global burden of disease due to SLT use, this review did not adhere to strict criteria for the definition of oral cancer, and hence also included studies that had data for cancers other than at oral sites such as the oropharynx^27^. Another global review that included only oral sites gave significant cancer risks^20^.

The present review compiles the regional risk estimates of various earlier reviews according to WHO regions. Regional risk estimates were different across the globe. Both of the studies that gave global estimates also provided regional estimates for SEAR, EMR, and AMR. Asthana et al.^20^ provided estimates for WPR not given by Siddiqi et al.^27^. Other reviews gave risk estimates for only one or two regions. SEAR and AMR had the highest risk, because of the high production of tobacco and use in these regions along with the high levels of carcinogens in the used SLT. AMR did not have a significant estimate like SEAR due to the variation in frequency and intensity of SLT use. Only Lee et al.^24^ gave significant estimates for AMR because most of the included studies were not adjusted for smoking and other potential confounding variables. For the India region, risk was higher compared to the global level, demonstrating the alarming rate at which the prevalence of oral cancer is increasing in India. High tobacco production and usage in India are the causes of the increased prevalence. Petti et al.^25^ showed the high prevalence of oral cancer associated with the use of SLT in WPR. The higher estimates were because the reviewers took smoking, drinking, and betel quid chewing into consideration as the covariates. AMR and EUR showed the least risk to oral cancer because of the low level of TSN and pH of the SLT used. Asthana et al.^20^ stated that the reason for low-risk estimates from Europe and the Americas might be due to difference in frequency and intensity of use and variation in SLT product type. Risk estimates for the India region were higher compared to the global level.

Most of the earlier reviews talked about either one or two specific SLT products or gave a combined risk. Only one recent review gave estimates on various SLT products^20^. The overview from individual systematic review estimates clearly indicates the potential specific risk associated with different type of SLT. When considering SLT as a whole, all reviews gave significant estimates, except Siddiqi et al.^27^ because their study had limitations in the included studies. Gutkha, paan/betel quid with tobacco, and chewing tobacco had the highest risk estimates within the type-specific SLT. Betel nut is a renowned carcinogen and is associated with a higher risk of oral cancer with or without tobacco^21-22^. Paan/betel quid and Gutkha contain betel nut, which explains the reason for the higher risk of oral cancer compared to others, due to the synergistic effects of various SLT products. Snus, nasal snuff, and snuff (unidentified), had lower risk estimates compared to others, because of the frequency and intensity of their use and low levels of carcinogens. Moist snuff used in Sweden was least toxic due to an improved manufacturing procedure and processing^35^. Our finding is that there is a higher risk of oral cancer associated with chewing tobacco products than non-chewing tobacco products.

Females were more prone to oral cancer associated with SLT than males. The risk was nearly three times higher in females compared to males. According to a review, the reason is not yet clear and requires further investigation^20^. According to Guha et al.^21^ it is possible that women chew more per day. Another explanation is that the relative risk may appear higher in women because they have a lower background risk for oral and oropharyngeal cancers than men, which is plausible since smoking and alcohol rates are much lower in women than in men in South Asia^2^.

Pooled analysis of case-control studies had higher odds ratios than cohort and combined (both case-control and cohort studies in pooled analysis); the ratio of studies that gave significant estimates for cohort and case-control was 1:3 and 4:5, respectively. Two studies conducted meta-analysis after 1990 and before 1990, separately. Studies conducted post-1990 showed lower risk estimates (Lee et al.^24^ and Asthana et al.^20^), possibly because of the interventions for improved tobacco quality and better analysis of data. Three out of four studies, which performed analysis for both cohort and case-control combined, gave significant estimates, except Siddiqi et al.^27^. There could be many reasons for such estimates. First, it might be the restriction of studies to countries for which reliable prevalence data and reliable risk data for diseases were available. Second, it was most likely the unavailability of country-specific data. These limitations made the estimates of less importance. The findings of the systematic review of Siddiqi et al.^27^ also concluded that 85% of deaths due to SLT-associated cancer were within South Asia, which might contradict the non-significant associations obtained in their analysis. There are various systematic reviews showing an association between SLT use and oral cancer but were not included in this review because they did not satisfy our inclusion criteria. They have similar and contrasting results with our study. They performed analyses for American, European, and Indian Subcontinent, populations^33,34,36^.

As anticipated, there were differential quality findings on systematic review studies that were evaluated by the AMSTAR 2 and CASP tools. AMSTAR 2 and CASP have made meta-analysis an essential aspect of the analysis making it both a qualitative and quantitative analysis. Thus, the scoring of the systematic reviews without meta-analysis declined proportionately more than those with meta-analysis. AMSTAR 2 is more accurate and gives high score on protocol registration, properly defined inclusion and exclusion criteria, and assessment of ROB. There are some reviews that performed the quality assessment of included studies but had uncertainty about inclusion of all relevant studies^23,30^.

### Limitations

A limitation of the present review of reviews is that it could not provide the pooled risk estimates in the situations with multiple reviews, because of the duplicity of the included studies. The pooled analysis in such conditions would not be appropriate. The range is vast for some attributes and thus would pose difficulty for the policymakers. It does not provide the exact weights of threat posed by SLT on these attributes. The review provides the range of risk estimates by accumulating effect measures data from various reviews.

## CONCLUSIONS

The strong relation between SLT use and oral cancer is evident and confirmed as observed in this review. The variability was present in oral cancer and SLT association across geographical regions. Gender and type-specific SLT-use differentials provide additional strong evidence. There is an immediate need to frame policies and strategies for the cessation of SLT use.

## References

[1] National Cancer Institute of National Institutes of Health Smokeless Tobacco and Cancer.

[2] National Cancer Institute, Centers for Disease Control and Prevention Smokeless Tobacco and Public Health: A Global Perspective.

[3] Food and Agriculture Organization of the United Nations FAOSTAT: Tobacco, unmanufactured.

[4] World Health Organization WHO Framework Convention on Tobacco Control.

[5] Ministry of Health and Family Welfare (2003). COTPA 2003 and Rules made there under.

[6] Sinha DN, Rizwan SA, Aryal KK, Karki KB, Zaman MM, Gupta PC (2015). Trends of smokeless tobacco use among adults (aged 15-49 years) in Bangladesh, India and Nepal. Asian Pac J Cancer Prev.

[7] World Health Organization (2017). Recommendation on smokeless tobacco products.

[8] Lipari RN, Van Horn SL (2013). Trends in Smokeless Tobacco Use and Initiation: 2002 to 2014. The CBHSQ Report.

[9] Niaz K, Maqbool F, Khan F, Bahadar H, Ismail Hassan F, Abdollahi M (2017). Smokeless tobacco (paan and gutkha) consumption, prevalence, and contribution to oral cancer. Epidemiol Health.

[10] World Health Organization (2008). WHO Report on the Global Tobacco Epidemic.

[11] Edge SB, Compton CC (2010). The American Joint Committee on Cancer: the 7th Edition of the AJCC Cancer Staging Manual and the Future of TNM. Ann Surg Oncol.

[12] American Cancer Society Survival Rates for Oral Cavity and Oropharyngeal Cancer.

[13] Rao SVK, Mejia G, Thomson KR, Logan R (2013). Epidemiology of Oral Cancer in Asia in the Past Decade- An Update (2000-2012). Asian Pac J Cancer Prev.

[14] Mallath MK, Taylor DG, Badwe RA (2014). The growing burden of cancer in India: epidemiology and social context. Lancet Oncol.

[15] World Health Organization International Agency for Research on Cancer.

[16] PRISMA PRISMA 2009 checklist.

[17] Critical Appraisal Skills Programme CASP Systematic Review Checklist.

[18] Gates A, Gates M, Duarte G (2018). Evaluation of the reliability, usability, and applicability of AMSTAR, AMSTAR 2, and ROBIS: protocol for a descriptive analytic study. Systematic Reviews.

[19] Shea BJ, Reeves BC, Wells G (2017). AMSTAR 2: a critical appraisal tool for systematic reviews that include randomised or non-randomised studies of healthcare interventions, or both. BMJ.

[20] Asthana S, Labani S, Kailash U, Sinha DN, Mehrotra R (2019). Association of Smokeless Tobacco Use and Oral Cancer: A Systematic Global Review and Meta-Analysis. Nicotine Tob Res.

[21] Guha N, Warnakulasuriya S, Vlaanderen J, Straif K (2014). Betel quid chewing and the risk of oral and oropharyngeal cancers: A meta-analysis with implications for cancer control. Int J Cancer.

[22] Gupta B, Johnson NW (2014). Systematic review and meta-analysis of association of smokeless tobacco and of betel quid without tobacco with incidence of oral cancer in South Asia and the Pacific. PLoS One.

[23] Khan Z, Tönnies J, Müller S (2014). Smokeless tobacco and oral cancer in South Asia: A systematic review with meta-analysis. J Cancer Epidemiol.

[24] Lee PN, Hamling J (2009). Systematic review of the relation between smokeless tobacco and cancer in Europe and North America. BMC Medicine.

[25] Petti S, Masood M, Scully C (2013). The Magnitude of Tobacco Smoking-Betel Quid Chewing-Alcohol Drinking Interaction Effect on Oral Cancer in South-East Asia. A Meta-Analysis of Observational Studies. PLoS One.

[26] Prasad JB, Dhar M (2018). Risk of major cancers associated with various forms of tobacco use in India: a systematic review and meta-analysis. J Public Health.

[27] Siddiqi K, Shah S, Abbas SM (2015). Global burden of disease due to smokeless tobacco consumption in adults: Analysis of data from 113 countries. BMC Med.

[28] Sinha DN, Abdulkader RS, Gupta PC (2016). Smokeless tobacco-associated cancers: A systematic review and meta-analysis of Indian studies. Int J Cancer.

[29] Awan KH, Patil S (2016). Association of smokeless tobacco with oral cancer – evidence from the South Asian Studies: A systematic review. J Coll Physicians Surg Pak.

[30] Khan Z, Suliankatchi RA, Heise TL, Dreger S (2019). Naswar (Smokeless Tobacco) Use and the Risk of Oral Cancer in Pakistan: A Systematic Review With Meta-Analysis. Nicotine Tob Res.

[31] Gupta S, Gupta R, Sinha DN, Mehrotra R (2018). Relationship between type of smokeless tobacco & risk of cancer: A systematic review. Indian J Med Res.

[32] Critchley JA, Unal B (2003). Health effects associated with smokeless tobacco: A systematic review. Thorax.

[33] Wyss AB, Hashibe M, Lee YA (2016). Smokeless tobacco use and the risk of head and neck cancer: Pooled analysis of US studies in the INHANCE consortium. Am J Epidemiol.

[34] Weitkunat R, Sanders E, Lee PN (2007). Meta-analysis of the relation between European and American smokeless tobacco and oral cancer. BMC Public Health.

[35] Rodu B, Jansson C (2004). Smokeless tobacco and oral cancer: A review of the risks and determinants. Crit Rev Oral Biol Med.

[36] Boffetta P, Hecht S, Gray N, Gupta P, Straif K (2008). Smokeless tobacco and cancer. Lancet Oncol.

