# Supplementary material for: Association of smokeless tobacco with oral cancer: A review of systematic reviews
Source: Tob Prev Cessat. 2019 Oct 8;5:34. doi: 10.18332/tpc/112596 (PMC7205108; doi:10.18332/tpc/112596)
Supplement: Supplementary file 1 [file TPC-5-34-s1.pdf]

Web Table 1: Qualitative summary of included systematic reviews

Citations Details	The objective of included Review	Type of Review	Participant details	Study exposure	Number of databases sourced and searched	The data range of database searching	Confounders	The publication date range of studies included	Number of studies included type and country of origin of studies	Risk estimates (odds ratio or Relative risk)	Method of synthesis/ analysis employed to synthesize the evidence
Asthana S. et.al 2018 India (20)	The risk of oral cancer with the use of smokeless tobacco products	Systematic review and meta-analysis	Global population (M+F)	Pan tobacco/ areca nut + lime + tobacco, oral snuff, Snus/moist snuff, Gutkha, Manipuri, nasal snuff/dipping	(4) PubMed Indmed EMBASE Google Scholar	4846	Smoking	1960-2016	37 Case-Control, Cohort (SEAR, EUR, EMR, AMR	Odds ratio	Odds ratio [random effects], heterogeneity, publication bias
Guha N. et.al 2014 France (21)	Risk of oral and oropharyngeal cancer associated with betel quid	Systematic reviews and meta-analysis	Indian Subcontinent (M+F)	Betel quid	(3) PubMed MEDLINE IARC Monographs	NA	Alcohol Smoking	1933-2013	50 Cohort (8) Case-control (49) Taiwan 17 India32 Thailand 1 China 17 Pakistan 3 Srilanka 2	Odds' ratio Relative risk	Meta relative risk using the random-effects model, heterogeneity, PAF
Gupta B. et.al 20 Nov 2014 Australia (22)	Association between the use of oral smokeless tobacco in any form, of betel quid without tobacco and of areca nut with oral cancer in South Asia and the Pacific	Systematic Review and Meta-analysis	South Asia and the Pacific Region (M+F)	Smokeless Tobacco (NOS), Betel quid with Tobacco	(3) PubMed CINAHL Cochrane	3865 + 20 (ref.)	Smoking Alcohol Others*	Case-Control 1959-2012 Cohort 2008-2011	19 Case Control (15) Cohort (4) (India [11], Pakistan [1], Taiwan [4], Papua New Guinea [1])	Odds Ratio Relative Risk,	Adjusted odds ratio with 95% CI using crude effect, heterogeneity using Higgins' H and I^2 statistics, Funnel plots and Egger's test were used to evaluate Publication bias.

Khan Z. et.al 2014 Germany (23)	Epidemiological association of SLT in observational studies	Systematic review and meta-analysis	South Asians (M+F)	Pan, Gutkha, Betel Nut, Areca Nut, smokeless tobacco	(2) MEDLINE ISI Web of knowledge	735	Smoking Alcohol	1989-2013	21 Case-control [18], Cohort [3] (India 19, Pakistan 2)	Odds ratio	Odds ratio [with inverse variance method using fixed and random effect method], heterogeneity
Lee P.N et.al 2009 UK (24)	To carry out a comprehensive review of the available epidemiological evidence in Western countries relating ST to cancer	Systematic review and meta-analysis	Europe and North Americans (M+F)	Smokeless tobacco, Snuff, Chewing Tobacco	(1) MEDLINE	690	Smoking	1920-2008	89 Case-control [80], Cohort [9] USA [62] Sweden [13] Canada [3] Denmark [3] UK [3] Brazil [1] Norway [2] Rico [1]	Odds ratio	Odds ratio [random effects], heterogeneity, publication bias
Petti S. et.al 2013 Italy (25)	Assess the magnitude of smoking-drinking-chewing interaction effect on oral cancer	Systematic reviews and meta-analysis	South East Asia M+F	Betel Quid	(6) MEDLINE PubMed Ovid Scopus Google Scholar, Reference	4417	Smoking Alcohol	1989-2011	14 Case-control India7 Taiwan7	Odds ratio	Pooled odds' ratio
Prasad J.B. et.al 2018 India (26)	Different cancer sites associated with various forms of tobacco	Systematic review and meta-analysis	Indian (M+F)	Chewing Tobacco	(3) Scopus, Science Direct database, Google Scholar	524	Smoking	1971-2015	22 Case-Control (India)	Odds ratio	Random effect odds ratio, Heterogeneity
Siddiqui K. et.al 2015 UK (27)	Global estimates of the burden of disease due to the consumption of smokeless tobacco by adults.	Systematic review and meta-analysis	Global Population (M+F)	Smokeless tobacco with/without tobacco, chewing tobacco, snuff, snus, gutkha, nass,	(23) **P6	6678	Smoking Alcohol	1952-2012	33 of 39 Cohort [5] Case-control [22] India [22] Pakistan [2] Sweden [4] Norway [1] USA [2]	Odds ratio	Random effect odds ratio, heterogeneity

Sinha D.N et. Al<br>7/10/15<br>India<br>(28)	Effect of smokeless tobacco use on cancer incidence among adults in INDIA.	Systematic review and Meta-analysis	Indian population (M+F);<br>Age (30-50yrs)	Smokeless tobacco	(10)<br><br>*A6	875 in PubMed and 4 in other sources	Smoking<br>Alcohol<br>Others*	1955-2015	25 studies, (India)<br>Case-Control (23)<br>Cohort (2)	Odds ratio	Odds ratio using the random effect model, heterogeneity test using I^2 statistics, publication bias,
Habib K. et. Al<br>2016<br>Saudi Arabia<br>(29)	South Asian studies to assess the association of SLT<br>and oral cancer	Systematic Review	South Asians (M+F)	Betel Quid, Chewing Tobacco	(4)<br>PubMed, Medline, EMBASE<br>ISI Web of Science	784	Smoking<br>Alcohol	1980-2015	21<br>Case-Control (18)<br>Cohort (30)<br>India (19)<br>Pakistan (2)	Odds Ratio	No meta-analysis conducted
Khan Z. et.al<br>2017<br>Pakistan<br>(30)	*Naswar* as carcinogenic	Systematic review and meta-analysis	Pakistan residents (M+F)	Naswar	(4)<br>MEDLINE<br>PubMed,<br>Science Citation Index,<br>WHO index for Eastern Mediterranean region	597	Smoking<br>Alcohol	1977-2017	6<br>Case-control (Pakistan)	Odds Ratio	Fixed effect odds ratio, Heterogeneity, PAF
Gupta S. et.al<br>07/18<br>India)<br>(31)	Cancer occurrence as well<br>as mortality risk in users of SLT products	Systematic Review	Global Population (M+F)	Smokeless tobacco	(2)<br>PubMed and Google Scholar	4470	Smoking<br>Alcohol<br>Others*	1985-2018	33 of 80<br>Cohort (5),<br>Case-Control(28)<br>SEAR (22)<br>EUR (3)<br>AMR (1<br>EMR (5)<br>AFR (2)	Odds Ratio	No meta-analysis conducted

*A6: databases such as PubMed, IndMED, Google Scholar, reports of the WHO, South-East Asia Region, CDC tobacco reports, MOHFW, India reports, Web of science, Science Citation Index, WHO Index Medicus of the South-East Asian Region and Open Grey.
**P6: Search engines included Medline, Embase, Web of Science, CINAHIL, Pakmed, Informit, Ingenta Connect, Global Health, AJLOL African Journals Online, Airiti Inc, Academic Search, Pubget, OALster, IndMED, LILACS and Cochrane Database were used. Moreover, Google Scholar, Pubmed Database (January 1946 - February 2015) and key websites such as (World Health Organisation (WHO), Action on Smoking and Health (ASH), UK; Action on Smoking and Health (ASH), USA; National Institutes of Health (NIH), and Centres for Disease Control and Prevention (CDC) was also searched.
*Others: Other factors include age, gender, socioeconomic status, income, education, occupation, religion, residence, body mass index, diet pattern and genotype
#M+F: Males + Females

Web Table 2: Risk of SLT use on ORAL CANCER- A Subgroup analysis (Summaries of estimates from various systematic reviews)

STUDY TYPE			
**COHORT**	Asthana S. et.al 2018 (20) #	2.32 (0.91, 5.94)	91% (<0.001)
	Lee P.N. et.al 2009 (24) #	1.32 (0.65, 2.68)	29.8%
	Guha N. et.al 2014 (21) #	15.87 (6.56, 38.40)	79.8%
**CASE-CONTROL**	Asthana S. et.al 2018 (20)	3.66 (2.83, 4.74)	97% (<0.001)
	Lee P.N. [1] et.al 2009 (24)	2.38 (1.87, 3.04)	29.8%
	Lee P.N. [2] et.al 2009 (24)	1.0 (0.83–1.20)	29.8%
	Guha N. et.al 2014 (21)	7.23 (4.96, 10.56)	96.6%
	Prasad J.B. 2018 (26)	6.59 (5.18-8.39)	74.9% (0.001)
**COMBINED**	Sinha D.N. et. al 2015 (28)	5.55 (5.07-6.07)	95% (<0.001)
	Asthana S. et.al 2018 (20)	3.53 (2.75, 4.51)	97% (<0.001)
	Petti S. et.al 2013 (25)	7.90 (6.71, 9.30)	NA
	Siddiqui K. et.al 2015 (27)	3.43 (0.70, 1.28)	0% (<0.001)

values of relative risk

Lee P.N.[1] et.al 2009 (24): Odds' ratio of studies from 1920-1988

Lee P.N. [2] et.al 2009 (24): Odds ratio of studies after 1990

Web Table 3: Scoring of reviews according to CASP tool

*Score is low due to nature of the study being only systematic reviews

References	Q1.	Q2.	Q3.	Q4.	Q5.	Q6.	Q7.	Q8.	Q9.	Q10.	SCORE
(20)	yes	yes	yes	No	Yes	yes	yes	yes	Yes	yes	9
(21)	yes	Yes	Yes	No	Yes	Yes	Yes	Yes	Yes	Yes	9
(22)	Yes	Yes	Can't tell	No	Yes	yes	yes	yes	Yes	yes	8
(23)	yes	yes	Cant' tell	yes	Yes	yes	yes	yes	Yes	yes	9
(24)	yes	yes	yes	no	Yes	yes	yes	yes	Yes	yes	9
(25)	yes	yes	Can't tell	Can't tell	Yes	Yes	Yes	Yes	Yes	Yes	8
(26)	yes	yes	yes	no	Yes	yes	Yes	Yes	Yes	Yes	9
(27)	Yes	Yes	Yes	No	Yes	Yes	Yes	Yes	Yes	Yes	9
(28)	Yes	Yes	Yes	no	Yes	Yes	Yes	Yes	Yes	Yes	9
(29)	Yes	Yes	No	no	(Meta-analysis was not conducted)	(Meta-analysis was not conducted)	(Meta-analysis was not conducted)	yes	No	yes	4*
(30)	yes	yes	Can't tell	yes	Yes	yes	yes	Yes	Yes	yes	9
(31)	Yes	Yes	no	No	(Meta-analysis was not conducted)	(Meta-analysis was not conducted)	(Meta-analysis was not conducted)	Yes	No	Yes	4*

Q1. Did the review address a clearly focused question?
Q2. Did the authors look for the right type of papers?
Q3. Do you think all the important, relevant studies were included?
Q4. Did the review's authors do enough to assess quality of the included studies?
Q5. If the results of the review have been combined, was it reasonable to do so?
Q6. What are the overall results of the review?

Q7. How precise are the results?
Q8. Can the results be applied to the local population?
Q9. Were all important outcomes considered?
Q10. Are the benefits worth the harms and costs?

Web Table 4: Rating of Systematic Reviews According to AMSTAR 2

Q1. Did the research questions and inclusion criteria for the review include the components of PICO?

Q2. Did the report of the review contain an explicit statement that the review methods were established prior to the conduct of the review and did the report justify any

Reference	Ques 1	Ques 2	Ques 3	Ques 4	Ques 5	Ques 6	Ques 7	Ques 8	Ques 9	Ques 10	Ques 11	Ques 12	Ques 13	Ques 14	Ques 15	Ques 16	SCORE
20	Yes	Partial Yes	Yes	Partial Yes	Yes	Yes	Partial Yes	Yes	Yes	No	Yes	No	No	No	Yes	Yes	Low
21	Yes	Partial Yes	Yes	Partial Yes	Yes	Yes	No	Partial Yes	Yes	No	Yes	Yes	Yes	Yes	Yes	No	High
22	Yes	Partial Yes	Yes	Partial Yes	Yes	Yes	Yes	Yes	Yes	Yes	Yes	No	No	No	Yes	Yes	Low
23	Yes	Partial Yes	Yes	Partial Yes	Yes	Yes	Partial Yes	Partial Yes	Yes	No	Yes	Yes	Yes	No	No	Yes	Low
24	Yes	Yes	Yes	Partial Yes	No	No	Partial Yes	Yes	Yes	Yes	Yes	Yes	Yes	Yes	Yes	Yes	Moderate
25	Yes	Partial Yes	Yes	Partial Yes	Yes	Yes	Partial Yes	Yes	Yes	Yes	Yes	Yes	Yes	Yes	Yes	Yes	High
26	Yes	No	Yes	Partial Yes	No	No	Partial Yes	No	No	Yes	Yes	No	No	No	No	Yes	Critically Low
27	Yes	Partial Yes	Yes	Yes	Yes	Yes	Partial Yes	Yes	Yes	Yes	Yes	No	No	No	No	Yes	Critically Low
28	Yes	Yes	Yes	Yes	Yes	Yes	Partial Yes	Yes	Yes	Yes	Yes	Yes	Yes	Yes	Yes	Yes	High
29	Yes	No	Yes	Partial Yes	Yes	Yes	No	Yes	No	No	No Meta-Analysis Conducted	No Meta-Analysis Conducted	No	No	No Meta-Analysis Conducted	No	Critically Low
30	Yes	Yes	Yes	Partial Yes	Yes	Yes	No	Yes	Yes	Yes	No	No	No	No	No	Yes	Critically Low
31	Yes	No	Yes	Partial Yes	Yes	Yes	No	Partial Yes	No	Yes	No Meta-Analysis Conducted	No Meta-Analysis Conducted	No	No	No Meta-Analysis Conducted	Yes	Critically Low

significant deviations from the protocol?
Q3.Did the review authors explain their selection of the study designs for inclusion in the review?
Q4. Did the review authors use a comprehensive literature search strategy?
Q5. Did the review authors perform study selection in duplicate?
Q6. Did the review authors perform data extraction in duplicate?
Q7. Did the review authors provide a list of excluded studies and justify the exclusions?
Q8. Did the review authors describe the included studies in adequate detail?
Q9. Did the review authors use a satisfactory technique for assessing the risk of bias (RoB) in individual studies that were included in the review?
Q10. Did the review authors report on the sources of funding for the studies included in the review?
Q11. If meta-analysis was performed, did the review authors use appropriate methods for statistical combination of results?
Q12. If meta-analysis was performed, did the review authors assess the potential impact of RoB in individual studies on the results of the meta-analysis or other evidence synthesis?
Q13. Did the review authors account for RoB in primary studies when interpreting/discussing the results of the review?
Q14. Did the review authors provide a satisfactory explanation for, and discussion of, any heterogeneity observed in the results of the review?
Q15. f they performed quantitative synthesis did the review authors carry out an adequate investigation of publication bias (small study bias) and discuss its likely impact on the results of the review?
Q16. Did the review authors report any potential sources of conflict of interest, including any funding they received for conducting the review?

© 2019 Asthana S.
